# Supplementary material for: Smooth Interpolating Curves with Local Control and Monotone Alternating Curvature
Source: Comput Graph Forum. 2022 Oct 6;41(5):25–38. doi: 10.1111/cgf.14600 (PMC9827861; doi:10.1111/cgf.14600)
Supplement: Supplementary file 1 — Supplement Material [file CGF-41-25-s001.zip › Local-Smooth-Interpolating-MonoCurvature/extern/clothoids/submodules/Utils/docs/api-cpp/enum_a00083_1a3b83d0d73d49b5a75c3b92031f2c77ee.html]

Enum fgB — Utils v1.2

### Navigation

- index
- toc
- next
- previous
- Utils »
- C++ API »
- Enum fgB

# Enum fgB¶

- Defined in File rang.hxx

## Enum Documentation¶

enum rang::fgB¶
:   *Values:*

    enumerator black¶

    enumerator red¶

    enumerator green¶

    enumerator yellow¶

    enumerator blue¶

    enumerator magenta¶

    enumerator cyan¶

    enumerator gray¶

### Quick search

### Table of Contents

- rang - Colors for your Terminal
- Zstr
- C++ API

«
hide menu

menu
sidebar
»

### Navigation

- index
- toc
- next
- previous
- Utils »
- C++ API »
- Enum fgB

© Copyright 2021, Enrico Bertolazzi.
Created using Sphinx 4.2.0.
